# Data for iTRAQ-based quantitative proteomics analysis of *Brassica napus* leaves in response to chlorophyll deficiency

**DOI:** 10.1016/j.dib.2014.10.004

**Published:** 2014-11-06

**Authors:** Pu Chu, Gui Xia Yan, Qing Yang, Li Na Zhai, Cheng Zhang, Feng Qi Zhang, Rong Zhan Guan

**Affiliations:** a State Key Laboratory of Crop Genetics and Germplasm Enhancement, Nanjing Agricultural University, Nanjing 210095, China; b Nanjing Agricultural University, Jiangsu Collaborative Innovation Center for Modern Crop Production, Nanjing, Jiangsu, China

## Abstract

The essential pigment chlorophyll (Chl) plays important roles in light harvesting and energy transfer during photosynthesis. Here we present the data from a comparative proteomic analysis of chlorophyll-deficient *Brassica napus* mutant cde1 and its corresponding wild-type using the iTRAQ approach (Pu Chu et al., 2014 [1]). The distribution of length and number of peptides, mass and sequence coverage of proteins identified was calculated, and the repeatability of the replicates was analyzed. A total of 443 differentially expressed proteins were identified in *B. napus* leaves, including 228 down-accumulated proteins mainly involved in photosynthesis, porphyrin and chlorophyll metabolism, biosynthesis of secondary metabolites, carbon fixation and 215 up-accumulated proteins that enriched in the spliceosome, mRNA surveillance and RNA degradation.

## Specifications table

**Table:** 

Subject area	*Biology*
More specific subject area	*Plant proteomics*
Type of data	*Table, figure*
How data was acquired	*iTRAQ, mass spectroscopy, instrument including Shimadzu LC-20AB HPLC system, Shimadzu LC-20AD Nano-HPLC system, AB SCIEX TripleTOF 5600 system*
Data format	*analyzed*
Experimental factors	*Protein samples were reduced with 10* *mM DTT, alkylated with 55* *mM iodoacetamide, digested using sequencing grade trypsin and labeled using iTRAQ 8-plex kits according to the manufacturer’s protocol.*
Experimental features	*An equal amount of total proteins was prepared from leaf tissue of wild-type and cde1 Brassica napus plants. After iTRAQ labeling, strong cation exchange chromatography was performed. TripleTOF analysis was then conducted and the MS/MS data obtained were searched using MASCOT for protein identification. For protein quantification, a 1.2-fold cutoff in addition of a p-value<0.05 was set to determine differentially accumulated proteins. Functional analysis of proteins identified was conducted using GO, COG and KEGG database.*
Data source location	*Nanjing, China*
Data accessibility	*The data are with this article*

## Value of the data

• Comprehensive protein profiles and identified important proteins in response to chlorophyll deficiency.
• The data provide new insights into the regulation of chlorophyll biosynthesis and photosynthesis in higher plants.
• The findings may be applied to genetic engineering for high photosynthetic efficiency in crops.
• 145 low molecular weight proteins (Mr<10 kDa) and 521 high molecular weight proteins (Mr>100 kDa) were identified using iTRAQ strategy.

## 1 Data

**Fig. 1:**
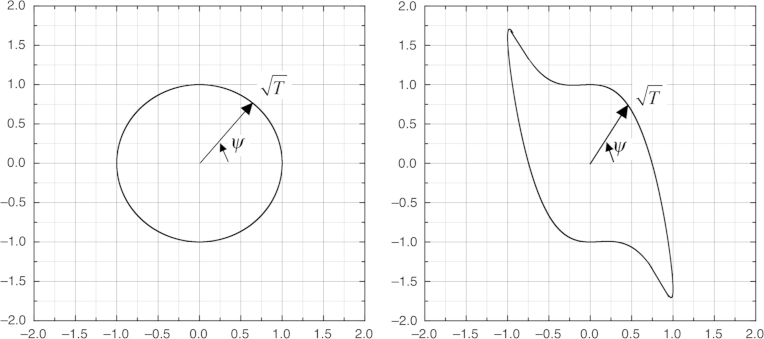
The distribution of length and number of peptides, mass and sequence coverage of proteins identified from iTRAQ proteomics.

Total proteins in *Brassica napus* leaves were extracted from the chlorophyll-deficient mutant *cde1* and the corresponding wild-type (WT) plants in three independent biological experiments using the iTRAQ approach. The distribution of lengths and numbers of peptides, mass and sequence coverage of proteins were shown in Fig. 1. Of the 5069 proteins identified in this study, over 60% of the proteins included at least two peptides and the sequence coverages of over 45% of the proteins were higher than 10%. Proteins with molecular masses too small or too large are difficult to be identified using traditional 2D gel technique. 145 low molecular weight proteins (Mr<10 kDa) and 521 high molecular weight proteins (Mr>100 kDa) were identified using iTRAQ strategy.

**Fig. 2:**
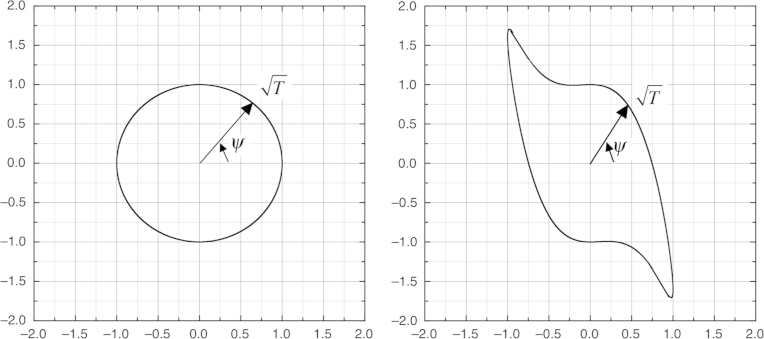
The repeatability of three replicates from the wild-type and *cde1* mutant. 1, 2, 3 represent three biological replicates of WT samples and 4, 5, 6 represent three biological replicates of *cde1* samples. The ratios of protein abundances for each protein in each comparison between biological replicates were calculated, and the “delta, error” in the abscissa represents the difference from the expected ratio of 1.

To demonstrate the repeatability of the replicates from the wild-type and *cde1* mutant, the protein abundances between various biological replicates were compared (Fig. 2). The results indicated that about 80% of the proteins showed less than 1.5-fold change between biological replicates.

Changes in the protein profile in response to *cde1* mutation were analyzed and 443 proteins showed a significant difference (*p*-value<0.05) with the false discovery rate (FDR) less than 5%. Among these proteins, 35, 116, and 228 proteins reproducibly decreased to less than 0.50-, 0.67-, and 0.83-fold, respectively (Supplementary Table 1). On the other hand, 33, 96, and 215 proteins increased by more than 2.0-, 1.5-, and 1.2-fold, respectively (Supplementary Table 2). Functional analyses of these differentially accumulated proteins were performed using Gene Ontology (GO), Clusters of Orthologous Groups of proteins (COG) and the Kyoto Encyclopedia of Genes and Genomes (KEGG) databases. The results showed that the down accumulated proteins included important enzymes in porphyrin and chlorophyll metabolism, carbon fixation and secondary metabolites biosynthesis. The abundance of photosynthetic proteins and proteins related to redox homeostasis was significantly reduced in the *cde1* mutant. Proteins that involved in spliceosome, mRNA surveillance, RNA degradation and protein modification were up accumulated in *cde1*, suggesting important roles of posttranscriptional RNA processing and posttranslational protein processing in the regulation of chlorophyll biosynthesis.

## 2 Experimental design, materials and methods

**Fig. 3:**
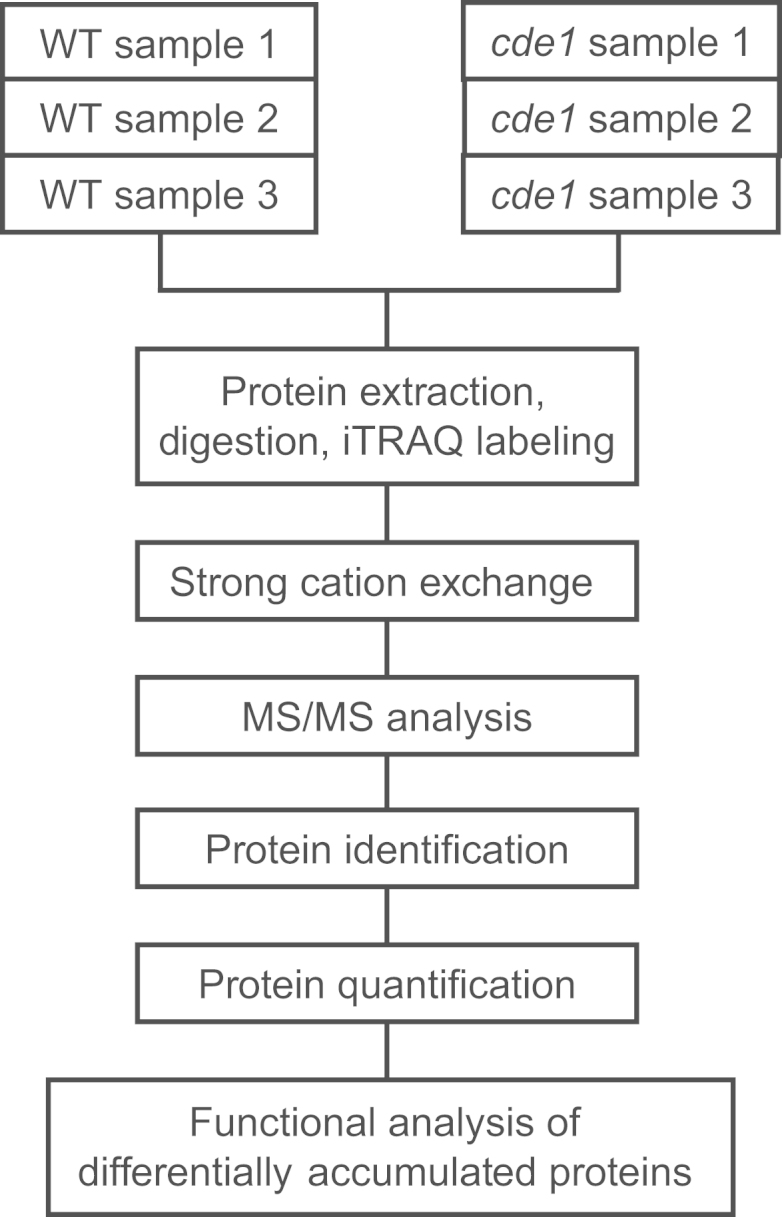
Flow chart of experimental design for the quantitative proteomics study.

Fig. 3 shows the experimental design used to gather the data presented here and in [1].

### 2.1 Plant materials

The *B. napus* chlorophyll-deficient mutant (*cde1*) was isolated from EMS-mutagenized *Brassica napus* cv. ‘NJ7982’ [2]. Plants were grown in the green house at the Nanjing Agriculture University Agronomy farm, Jiangpu, Nanjing, China. Fully expanded young leaves from 6-week-old plants were collected, immediately frozen in liquid nitrogen and kept at −80 °C until use.

### 2.2 Protein extraction, digestion, iTRAQ labeling

Total proteins were extracted from leaf tissue of wild-type (WT) and *cde1 B. napus* plants as previously described [3]. Three biological replicates were carried out for each sample. The Bradford method [4] determined the protein content. An equal amount of proteins was prepared for each biological replication. Protein samples were reduced with 10 mM DTT, alkylated with 55 mM iodoacetamide, digested using sequencing grade trypsin (Promega) at a ratio of 1:10 (w:w) for 12 h at 37 °C, and labeled using iTRAQ 8-plex kits (AB Sciex Inc., Framingham, MA, USA) according to the manufacturer’s protocol. The WT samples were labeled with iTRAQ tags 113, 114 and 115, and the mutant samples were labeled with tags 116, 117 and 118, respectively.

### 2.3 Strong cation exchange

After labeling, the samples were combined and lyophilized. The peptide mixture was dissolved in 4 mL strong cation exchange (SCX) buffer A (25% v/v acetonitrile, 25 mM NaH_2_PO_4_, pH 2.7). The peptides were fractionated on a Shimadzu LC-20AB HPLC system with an Ultremex SCX column (4.6×250 mm). Peptides were eluted at a flow rate of at 1 mL/min with elution buffer B (25% v/v acetonitrile, 25 mM NaH_2_PO_4_, 1 M KCl, pH 2.7). The absorbance at 214 nm was monitored and 20 fractions were collected. Samples of each fraction were dried and desalted before LC–ESI MS/MS analysis.

### 2.4 MS/MS analysis

Peptides of each fraction (5 μL injections) were resolved in solvent A (5% acetonitrile, 0.1% formic acid) and centrifuged at 20 000*g* for 10 min. The supernatant was separated using a Shimadzu LC-20AD Nano-HPLC system with a flow rate of 300 nL/min. Peptides were eluted by application of a linear gradient from 5% solvent B (95% acetonitrile v/v, 0.1% formic acid) to 35% solvent B over 35 min, followed by ramping up to 60% solvent B over 5 min, up to 80% in 2 min and maintained for 1 min; chromatographic conditions (5%) were restored in 1 min and equilibrated in solvent A for 10 min. For the TripleTOF analysis, a TripleTOF 5600 system was applied. Data were acquired using an ion spray voltage of 2.5 kV, nitrogen gas of 30 psi, nebulizer gas of 15 psi, and an interface heater temperature of 150 °C. The MS was performed in a high-resolution mode (>30 000 fwhm) for TOF MS scans. For information dependent data acquisition (IDA), survey scans were acquired in 250 ms, and as many as 30 product ion scans were collected if they exceeded a threshold of 120 counts per second (counts/s) and with a 2+ to 5+ charge-state. A sweeping collision energy setting of 35±5 eV, coupled with iTRAQ adjust rolling collision energy was applied to all precursor ions for collision-induced dissociation. Dynamic exclusion was set for 1/2 of peak width (15 s), and then the precursor was refreshed off the exclusion list. 5600 MSConverter was used to convert raw data files acquired from the Orbitrap into MGF files.

### 2.5 iTRAQ protein identification and quantification

For protein identification, MS/MS data were searched using MASCOT version 2.3.02 (Matrix Science, London, United Kingdom) against the ‘plant’ subset of the NCBI non-redundant sequence databases (released in April 2013, 1 495 260 entries). The search parameters were as follows: threshold set-off at 0.05 in the ion-score cutoff (with 95% confidence); MS/MS fragment ion mass tolerance of ±0.1 Da; enzyme specificity was set to trypsin with one missed cleavage; peptide tolerance was set at 10 ppm; fixed modifications of carbamidomethylation at Cys and iTRAQ 8plex at Lys and the N-terminal amino group of peptides; variable modifications of oxidation at Met, iTRAQ 8plex at Tyr, and glutamine as pyroglutamic acid; peptide change was set at Mr and monoisotopic mass was chosen; charge states of peptides were set to +2 and +3. Only peptides with significance scores greater than “identity_ score” were counted as identified. MASCOT analyzed three biological replicates of the iTRAQ data; only data with a FDR less than 5% were used for subsequent data analysis. Considering that multiple MS/MS spectra match to one peptide, normalization of the signal intensities of each MS/MS spectra was performed to find the most likely expression ratio for a peptide [5]. To demonstrate the repeatability of the replicates, the protein abundances between various biological replicates were compared and ratios for each protein in each comparison were compared with 1. The difference was plotted against the percentage of the proteins quantified. For quantitative changes, a 1.2-fold cutoff was set to determine up-accumulated and down-accumulated proteins, with a *p*-value<0.05 present in at least two replicates [6,7].

### 2.6 Bioinformatics analysis

Functional analysis of proteins identified was conducted using GO annotation (http://www.geneontology.org/) and proteins were categorized according to their biological process, molecular function and cellular localization [8]. The differentially accumulated proteins were further assigned to the COG database (http://www.ncbi.nlm.nih.gov/COG/) [9] and the KEGG database (http://www.genome.jp/kegg/pathway.html) [10,11].
